# Critical roles for the phosphatidylinositide 3-kinase isoforms p110β and p110γ in thrombopoietin-mediated priming of platelet function

**DOI:** 10.1038/s41598-018-37012-9

**Published:** 2019-02-06

**Authors:** Samantha F. Moore, Nina R. Smith, Thomas A. Blair, Tom N. Durrant, Ingeborg Hers

**Affiliations:** 1 School of Physiology, Pharmacology and Neuroscience, Biomedical Sciences Building, University of Bristol, Bristol, BS8 1TD UK; 2 Present Address: Harvard Medical School and Boston Children’s Hospital, Division of Hematology/Oncology, Boston, Massachusetts, United States

## Abstract

Thrombopoietin (TPO) enhances platelet activation through activation of the tyrosine kinase; JAK2 and the lipid kinase phosphatidylinositide 3-kinase (PI3K). The aim of our study was to identify the PI3K isoforms involved in mediating the effect of TPO on platelet function and elucidate the underlying mechanism. We found that p110β plays an essential role in TPO-mediated (i) priming of protease-activated receptor (PAR)-mediated integrin α_IIb_β_3_ activation and α-granule secretion, (ii) synergistic enhancement of PAR-mediated activation of the small GTPase RAP1, a regulator of integrin activation and (iii) phosphorylation of the PI3K effector Akt. More importantly, the synergistic effect of TPO on phosphorylation of extracellular-regulated kinase (ERK1/2) and thromboxane (TxA_2_) synthesis was dependent on both p110β and p110γ. p110β inhibition/deletion, or inhibition of p110γ, resulted in a partial reduction, whereas inhibiting both p110β and p110γ completely prevented the synergistic effect of TPO on ERK1/2 phosphorylation and TxA_2_ synthesis. The latter was ablated by inhibition of MEK, but not p38, confirming a role for ERK1/2 in regulating TPO-mediated increases in TxA_2_ synthesis. In conclusion, the synergistic effect of TPO on RAP1 and integrin activation is largely mediated by p110β, whereas p110β and p110γ contribute to the effect of TPO on ERK1/2 phosphorylation and TxA_2_ formation.

## Introduction

The endogenous myeloproliferative leukaemia protein (c-MPL) agonist; thrombopoietin (TPO) is a cytokine primarily involved in regulating platelet production, but it can also act as a platelet primer by enhancing platelet activation and function^1–3^. This cytokine can therefore play a pro-thrombotic and potentially pathogenic role in clinical conditions where it is found to be elevated. These conditions include altered bone marrow haematopoiesis/failure^4,5^, coronary artery disease^6^, acute angina^7^, sepsis^8^, inflammatory bowel disease^9^, burns patients^10^ and smokers^11^. Furthermore, TPO mimetics are used in the clinic to treat primary immune thrombocytopenia. These mimetics could potentially alter/restore platelet function causing either beneficial or detrimental effects not associated with the increase in platelet count, however information on this is still scarce and conflicting^12^. We and others have previously demonstrated that the mechanism by which TPO can enhance platelet activation is dependent on the activation of both the non-receptor tyrosine kinase janus kinase 2 (JAK2) and the lipid kinase phosphatidylinositide 3-kinase (PI3K)^1,13^.

Agonist-mediated activation of PI3K leads to the production of the second messenger molecule phosphatidylinositol (3,4,5)-trisphosphate (PIP3) and is well established in playing a vital role in supporting platelet activation and thrombus formation. The class I PI3Ks of which there are four catalytic isoforms; are responsible for the production of PIP3. All four of these isoforms are expressed in platelets with the p110β isoform being the most abundant and p110δ the least (β > γ > α > δ, based on the copy number analyses)^14,15^. The p110β isoform has been robustly demonstrated to play a dominant role in regulating platelet function through use of pharmacological agents and genetic models. The deletion of the p110β isoform or expression of a kinase dead form results in near ablation of both thrombin and convulxin-mediated production of PIP3 and in the phosphorylation of the PI3K effector Akt in response to thrombin, convulxin, collagen, adenosine diphosphate (ADP) and the TxA_2_ analogue U46619^16,17^. Furthermore, p110β has been demonstrated to support platelet function and thrombus formation after arterial injury but is not involved in regulating the haemostatic response^16^. In contrast, more minor roles have been reported for the other isoforms. The p110γ isoform appears to be primarily involved in regulating platelet response to ADP, with platelets deficient in p110γ having impaired aggregation in response to ADP and ablated ADP-mediated Akt phosphorylation^18,19^. The p110δ isoform plays a minor functional role in signalling downstream of GPVI and integrin α_IIb_β_3_^20^, whereas the p110α isoform has an unusual role in regulating the ability of platelet priming agents to enhance platelet function^21–24^.

The aim of this study was to investigate which PI3K isoforms can contribute to the priming effect of TPO on platelet function using a combination of pharmacological inhibitors and genetic mouse models. Here we demonstrate that the priming effect of TPO on integrin α_IIb_β_3_ activation and α-granule secretion is primarily mediated through the PI3K isoform p110β and involves the synergistic activation of the small GTPase RAP1. Furthermore, we have evidence that TPO/c-MPL in platelets can signal through the PI3K isoform p110γ; an isoform more commonly associated with being activated downstream of G-protein-coupled receptors (GPCR). Activation of this isoform in combination with p110β by TPO, is a requirement for the synergistic activation of the mitogen-activated protein kinase (MAPK) extracellular signal–regulated kinase (ERK1/2) and subsequent priming of TxA_2_ production.

## Results

### Thrombopoietin enhances platelet function through PI3-kinase

**Figure 1:**
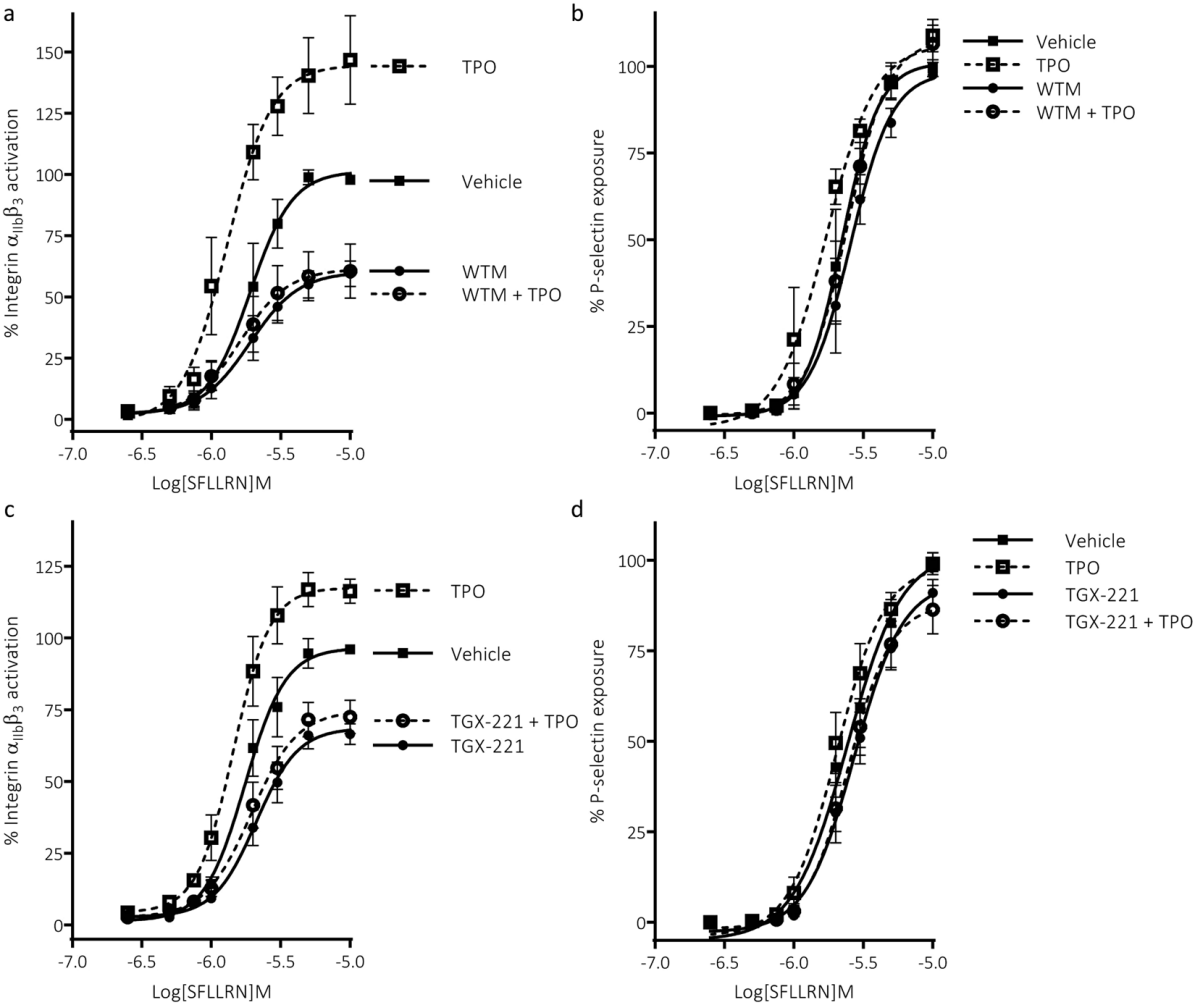
Enhancement of platelet function by TPO is supressed by the pan-PI3K inhibitor wortmannin and the p110β selective inhibitor TGX-221. (**a**) SFLLRN-mediated integrin α_IIb_β_3_ activation was enhanced by TPO (100 ng/ml, 5 min), resulting in a left shift of the curve (pEC50 = 5.73 ± 0.1 to 5.90 ± 0.1, n = 4 ± s.e.m, p < 0.001) and an increase in the maximal response (p < 0.01). Pre-incubation of platelets with the pan-PI3K inhibitor wortmannin (WTM) ablated the left shift in the curve (pEC50 = 5.72 ± 0.09 to 5.77 ± 0.09, n = 4 ± s.e.m, p > 0.05) and the increase in the maximal response (p > 0.05). (**b**) SFLLRN-mediated α-granule secretion (P-selectin exposure) was moderately enhanced by TPO (100 ng/ml, 5 min), resulting in a left shift of the curve (pEC50 = 5.66 ± 0.08 to 5.79 ± 0.07, n = 3 ± s.e.m, p < 0.05) but not an increase in the maximal response (p > 0.05). Pre-incubation with WTM ablated the left shift in the curve (pEC50 = 5.60 ± 0.07 to 5.64 ± 0.07, n = 3 ± s.e.m, p > 0.05). (**c**) SFLLRN-mediated integrin α_IIb_β_3_ activation was enhanced by TPO (100 ng/ml, 5 min), resulting in a left shift of the curve (pEC50 = 5.71 ± 0.06 to 5.80 ± 0.07 n = 8 ± s.e.m, p < 0.001) and an increase in the maximal response (p < 0.001). Pre-incubation of platelets with the PI3K p110β selective inhibitor TGX-221 (200 nM, 15 min) ablated the left shift in the curve (pEC50 = 5.66 ± 0.06 to 5.69 ± 0.06, n = 8 ± s.e.m, p > 0.05) and the increase in the maximal response (p > 0.05). (**d**) SFLLRN-mediated α-granule secretion (P-selectin exposure) was moderately enhanced by TPO (100 ng/ml, 5 min), resulting in a left shift of the curve (pEC50 = 5.60 ± 0.06 to 5.70 ± 0.06, n = 8 ± s.e.m, p < 0.01) but not an increase in the maximal response (p > 0.05). Pre-incubation with TGX-221 ablated the left shift in the curve (pEC50 = 5.60 ± 0.06 to 5.60 ± 0.06, n = 8 ± s.e.m, p > 0.05). Curves were fitted for each individual donor and curve parameters calculated using a four-parameter logistic equation (GraphPad Prism 7.0). For displayed graphs, concentration-response curves from different donors were pooled after normalising them to the maximal response (curve top) obtained in the vehicle-control samples. Statistical testing of curve parameters was performed using 2-way ANOVAs with Bonferroni’s multiple comparisons test: (Variable 1 = TPO, Variable 2 = Inhibitor). Significance was determined when p < 0.05.

To confirm that TPO increases platelet function in a PI3K-dependent manner, we examined PAR-mediated (SFLLRN) integrin α_IIb_β_3_ activation and α-granule secretion in washed platelets pre-treated with TPO in the absence and presence of the pan-PI3K inhibitor wortmannin. TPO markedly enhanced PAR-mediated integrin α_IIb_β_3_ activation, resulting in a left-shift of the concentration-response curve (pEC_50_ = 5.73 ± 0.1 to 5.90 ± 0.1, n = 4 ± s.e.m) and an increase in maximal integrin activation (Fig. 1a, Supplementary Fig. S1). TPO also induced alterations in PAR-mediated α-granule secretion, as measured by P-selectin exposure (Fig. 1b, Supplementary Fig. S1). The effect of TPO on α-granule secretion was muted compared to the effect on integrin α_IIb_β_3_ activation, the curve was left-shifted (pEC50 = 5.66 ± 0.08 to 5.79 ± 0.07, n = 3 ± s.e.m), but maximal P-selectin exposure was unaltered. Wortmannin treatment reduced maximal PAR-mediated integrin activation by ~40%, whereas α-granule secretion was minimally affected. Furthermore, the priming effects of TPO on both integrin α_IIb_β_3_ activation and α-granule secretion were prevented by pre-treating platelets with wortmannin (Fig. 1a,b). These results are in agreement with previous findings^13,25,26^.

### Enhancement of integrin α_IIb_β_3_ activation and α-granule secretion are dependent on p110β

The class IA PI3K isoforms (catalytic subunits; p110α, β and δ) are known to be activated downstream of tyrosine-kinase associated receptors via the recruitment of p85–p110 complexes and relief of p85 inhibition on the p110 catalytic subunits. Of these isoforms p110β is the most abundant^14,15^ in platelets and has been demonstrated to play an integral role in platelet activation^27^ and in supporting platelet priming in combination with the p110α isoform^21,24^. Pre-treatment of platelets with the p110β inhibitor TGX-221 had a similar effect as wortmannin on integrin α_IIb_β_3_ activation and α-granule secretion (Fig. 1c,d). Maximal PAR-mediated integrin α_IIb_β_3_ activation was reduced by ~30%, whereas α-granule secretion was unaltered; results in line with previous findings^28^. Furthermore, TGX-221 suppressed TPO-mediated enhancement of integrin α_IIb_β_3_ activation and α-granule secretion (Fig. 1c,d). In the presence of TGX-221, TPO failed to left-shift PAR-mediated integrin α_IIb_β_3_ activation (pEC50 = 5.66 ± 0.06 to 5.69 ± 0.06, n =8  ± s.e.m) or α-granule secretion (pEC50 = 5.60 ± 0.06 to 5.60 ± 0.06, n = 8 ± s.e.m) or increase maximal integrin activation.

**Figure 2:**
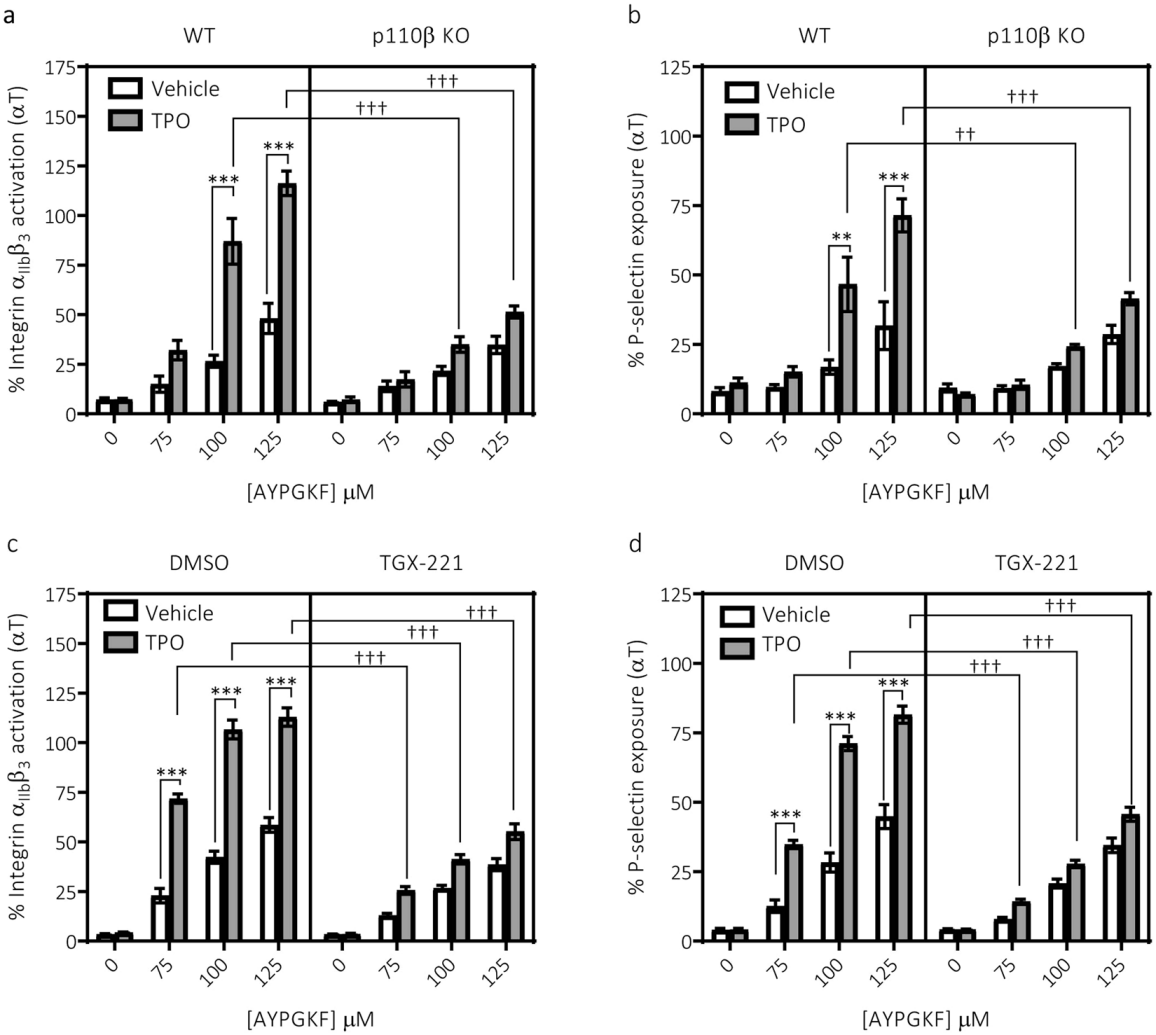
Enhancement of mouse platelet function by TPO is supressed by either deletion or inhibition of PI3K p110β. (**a**) AYPGKF-mediated integrin α_IIb_β_3_ activation (n = 3 ± s.e.m) and (**b**) α-granule secretion was enhanced by TPO (100 ng/ml, 5 min) in platelets from wild-type control (WT, mixed C57BL/6J × 129 Sv) mice. This enhancement was markedly reduced in platelets from p110β knock-out (KO) mice. Similarly, (**c**) AYPGKF-mediated integrin α_IIb_β_3_ activation (n = 3 ± s.e.m) and (**d**) α-granule secretion was enhanced by TPO (100 ng/ml, 5 min) in platelets from wild-type (WT, C57BL/6J) mice in the absence but not presence of the PI3K p110β selective inhibitor TGX-221 (200 nM, 15 min). Data from individual experiments was pooled after normalising them to a maximum response evoked in an independent sample (wild-type/vehicle treated platelets stimulated with 0.1 U/mL thrombin). 2-way ANOVAs were performed with Bonferroni’s multiple comparisons test: (*Variable 1 = AYPGKF concentration, Variable 2 = TPO or ^†^Variable 1 = AYPGKF concentration, Variable 2 = Genotype/Inhibitor). Significance was determined when p < 0.05, *p < 0.05, **p < 0.01, ***p < 0.001.

To strengthen these findings, we aimed to determine the effect of genetic deletion of p110β on the priming effect of TPO on PAR-mediated integrin activation. As platelets are anucleate cells not easily amenable to genetic manipulation we employed the use of a transgenic mouse where p110β is knocked-out. We determined that TPO enhanced platelet functional responses in wild-type control mouse platelets (mixed C57BL/6J × 129 Sv) elicited by sub-maximal concentrations of the PAR-4 selective agonist; AYPGKF (in mouse platelets in contrast to human platelets, PAR-4 is the dominant thrombin receptor). Interestingly, functional responses in mouse platelets compared to human appeared to be more susceptible to enhancement by TPO. However, in support of our findings in human platelets, this enhancement was suppressed in platelets deficient in the p110β isoform (Fig. 2a,b). Similar but not identical findings were obtained when wild-type mouse platelets (C57/BL6) were treated with TGX-221 (Fig. 2c,d). Interestingly, TPO was still able to significantly enhance integrin α_IIb_β_3_ activation (Supplementary Fig. S1) in human platelets in the presence of PI3K isoform selective inhibitors for p110α, p110δ and p110γ, and in p110α-deficient mouse platelets^21^ (Supplementary Fig. S2). Together these results demonstrate that in human and mouse platelets p110β is the dominant PI3K isoform involved in regulating the priming effect of TPO.

### p110β regulates the ability of TPO to synergistically enhance RAP1 activation

**Figure 3:**
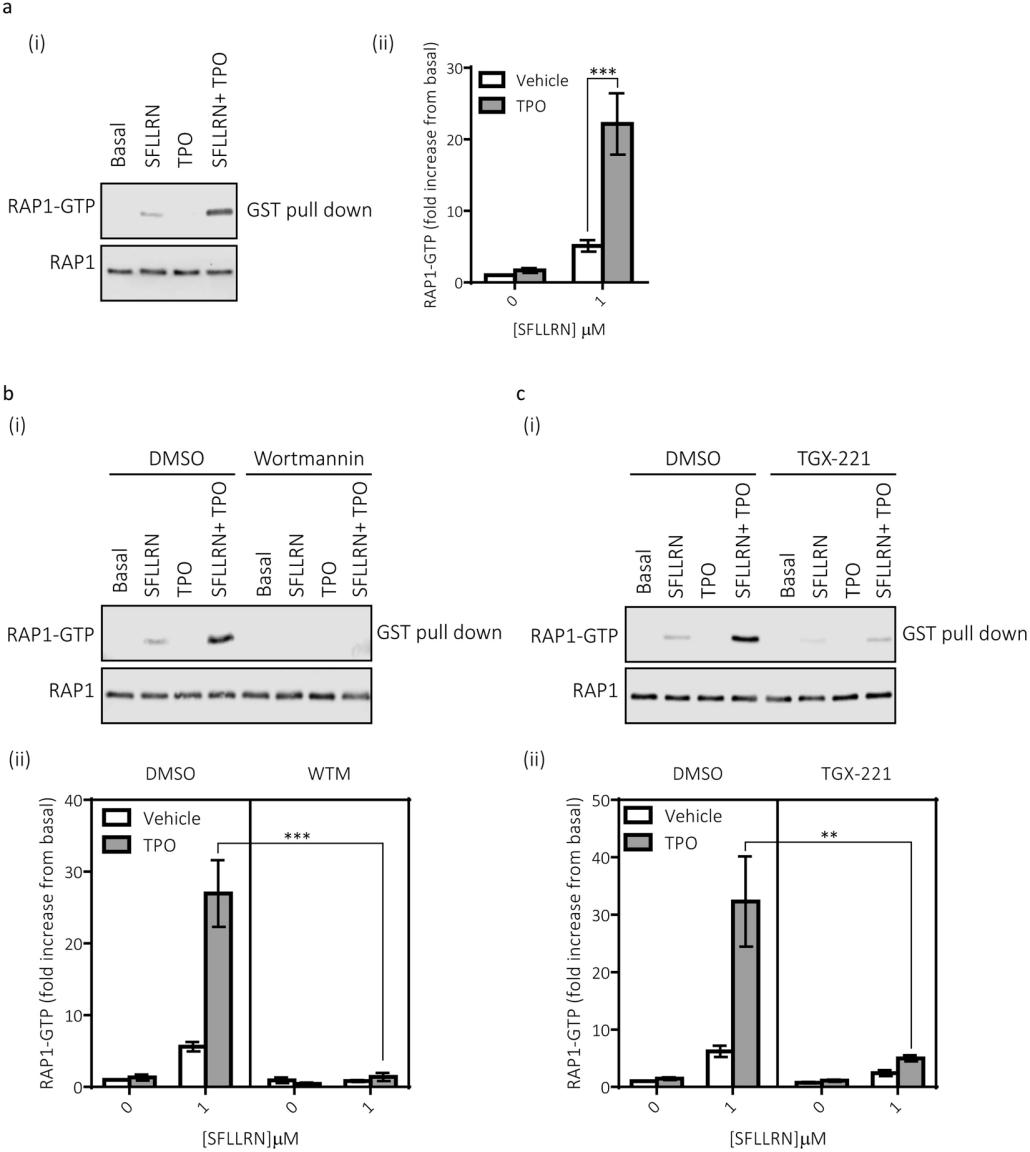
Critical role for PI3K p110β in the synergistic activation of the small GTPase RAP1. Washed human platelets were pre-incubated with TPO (100 ng/ml) for 5 min before stimulation with SFLLRN (1 μM, 5 min) in the absence or presence of indicated PI3K inhibitors. Platelets were lysed in RAP1 lysis buffer and incubated with the GST-tagged Rap binding domain of RAPGDS (GST-RALGDS-RBD), which specifically precipitates the active GTP-bound form of RAP1. Active RAP1 was identified by immunoblotting with an anti-RAP1 antibody. Matching whole cell lysates were run on different gels to determine the total amount of RAP1 (active and inactive) present. LI-COR® Image Studio (LI-COR®, Cambridge, UK) was used to create final images and to quantify bands as outlined in the Material and Methods. Full images and molecular weights from these experiments are presented in Supplementary Fig. S5. (**a**) Immunoblotting determined that TPO can synergistically enhance GTP loading of RAP1, with the combined action of TPO and SFLLRN on mediating RAP1 GTP loading being stronger than the sum of their effects (n = 9 ± s.e.m), 2-way ANOVA was performed with Bonferroni’s multiple comparisons test: (Variable 1 = SFLLRN, Variable 2 = TPO). (**b**) Pre-treatment of platelets with the pan PI3K inhibitor wortmannin (100 nM, 15 min) ablated the increase in RAP1-GTP (n = 4 ± s.e.m), 2-way ANOVA was performed with Bonferroni’s multiple comparisons test: (Variable 1 = SFLLRN, Variable 2 = WTM). (**c**) Furthermore, pre-treatment of platelets with the p110β inhibitor TGX-221 (200 nM, 15 min) also ablated the increase in RAP1-GTP (n = 9 ± s.e.m). 2-way ANOVA was performed with Bonferroni’s multiple comparisons test: (Variable 1 = SFLLRN, Variable 2 = TGX-221). Significance was determined when p < 0.05, *p < 0.05, **p < 0.01, ***p < 0.001.

The small G-protein RAP1 is a well-established critical common regulator of platelet function, with the deletion of the predominate isoform; Rap1b, resulting in impaired integrin α_IIb_β_3_ activation and granule secretion^29,30^. Here we demonstrate that TPO mediates a synergistic enhancement in RAP1 activation. Stimulation of platelets with a sub-maximal concentration of SFLLRN induced RAP1 guanosine-5′-triphosphate (GTP) loading above basal whereas TPO alone did not (Fig. 3a). Interestingly, SFLLRN in the presence of TPO resulted in a strong synergistic enhancement in RAP1 activation, with the combined action of TPO and SFLLRN stimulating a stronger response than the sum of their effects (Fig. 3a). In line with our findings from platelet activation, the generic PI3K inhibitor wortmannin (Fig. 3b) and the p110β inhibitor TGX-221 (Fig. 3b,c) blocked the synergistic enhancement in RAP1 activation. Although the interpretation of the wortmannin data may be confounded by the absence of RAP1 activation by SFLLRN alone, this was not the case for TGX-221 where SFLLRN-mediated RAP1 activation was still detectable (p < 0.01, n = 9). Together, this data therefore suggests that TPO enhances integrin α_IIb_β_3_ activation and α-granule secretion through p110β-mediated increases in RAP1 activation.

### A role for p110β in regulating TPO-mediated enhancement of PAR-mediated signalling events

**Figure 4:**
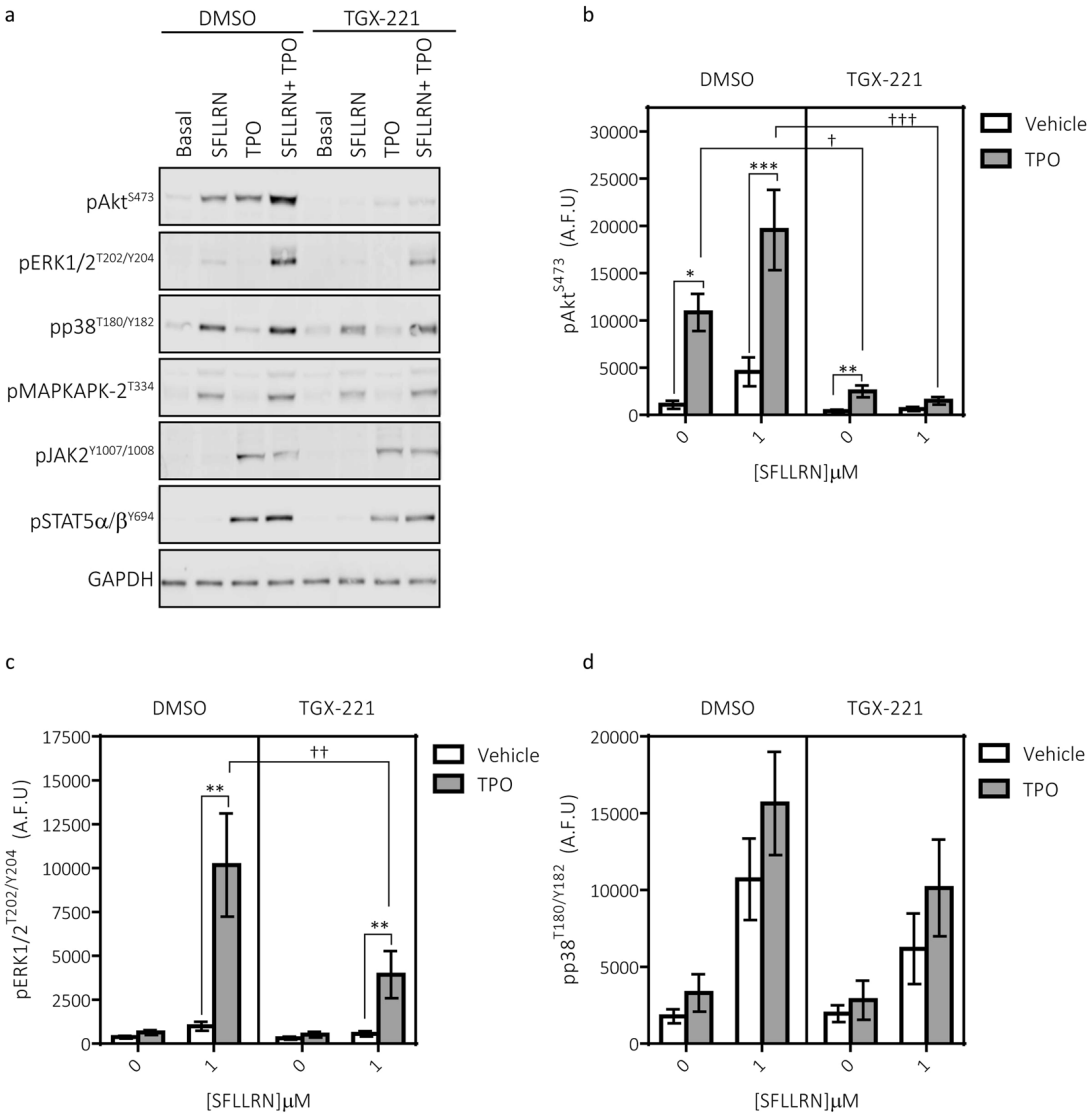
TPO enhances SFLLRN-mediated signalling events. Washed human platelets were pre-incubated with TPO (100 ng/ml) for 5 min before stimulation with SFLLRN (1 μM, 5 min) in the absence or presence of the p110β inhibitor TGX-221 (200 nM, 15 min). Platelets were lysed in 4x NuPAGE sample buffer containing 0.5M DTT and protein phosphorylation analysed by electrophoresis/immunoblotting. Samples were run on multiple gels for probing with multiple antibodies. For some experiments, gels and membranes were cut into two parts to be probed separately with different primary antibodies (e.g. pJAK2^Y1007/1008^ and pAkt^S473^, pSTAT5α/β^Y694^ and pERK^T202/Y204^). LI-COR® Image Studio (LI-COR®, Cambridge, UK) was used to create final images and to quantify bands as outlined in the Material and Methods. Full images and molecular weights from these experiments are presented in Supplementary Fig. S6. All samples in the panel figure shown are derived from the same donor/experiment. (**a**) Representative blot demonstrating that TPO can enhance phosphorylation of Akt^S473^, ERK1/2^T202/Y204^, p38^T180/Y182^ and its substrate MAPKAPK2^T334^. These enhancements were reduced by TGX-221. (**b**) TPO induces phosphorylation of Akt^S473^ and enhances SFLLRN-mediated phosphorylation. TPO-meditated effects on Akt^S473^ phosphorylation were reduced by TGX-221 (n = 9 ± s.e.m). (**c**) TPO did not evoke phosphorylation of ERK1/2^T202/Y204^ alone, however it induces a synergistic enhancement of SFLLRN-mediated phosphorylation which was reduced by TGX-221 (n = 9 ± s.e.m). (**d**) TPO in the absence and presence of TGX-221 induced subtle increases in SFLLRN-mediated phosphorylation of p38^T180/Y182^ (n = 5 ± s.e.m). Data are expressed as arbitrary fluorescence units (A.F.U, LI-COR® Image Studio). 2-way ANOVAs were performed with Bonferroni’s multiple comparisons test: (*Variable 1 = SFLLRN, Variable 2 = TPO or ^†^Variable 1 = SFLLRN, Variable 2 = TGX-221). Significance was determined when p < 0.05, *p < 0.05, **p < 0.01, ***p < 0.001.

The enhancement in platelet functional responses occurred with a concurrent enhancement in the activation of multiple signalling pathways (Fig. 4a). In line with previous findings, TPO induced the phosphorylation of the PI3K effector Akt and enhanced the phosphorylation of Akt induced by sub-maximal concentrations of SFLLRN (Fig. 4a,b). Akt phosphorylation by TPO alone or the combination of TPO and SFLLRN was almost ablated by the p110β inhibitor TGX-221. In contrast, TPO was unable to stimulate phosphorylation of the classical MAP kinases ERK1/2 but induced a striking synergistic enhancement of SFLLRN-mediated ERK1/2 phosphorylation (Fig. 4a,c). This synergistic enhancement was reduced but not abolished by TGX-221. SFLLRN-mediated phosphorylation of p38 and its substrate MAPKAPK-2 was subtlety increased in the presence of TPO, however this did not reach significance and was not altered by TGX-221 (Fig. 4a,d). Similar results were obtained using p110β-deficient platelets and mouse platelets pre-treated with TGX-221 (Supplementary Fig. S3).

### Synergistic phosphorylation of ERK1/2 is mediated by p110β and p110γ

**Figure 5:**
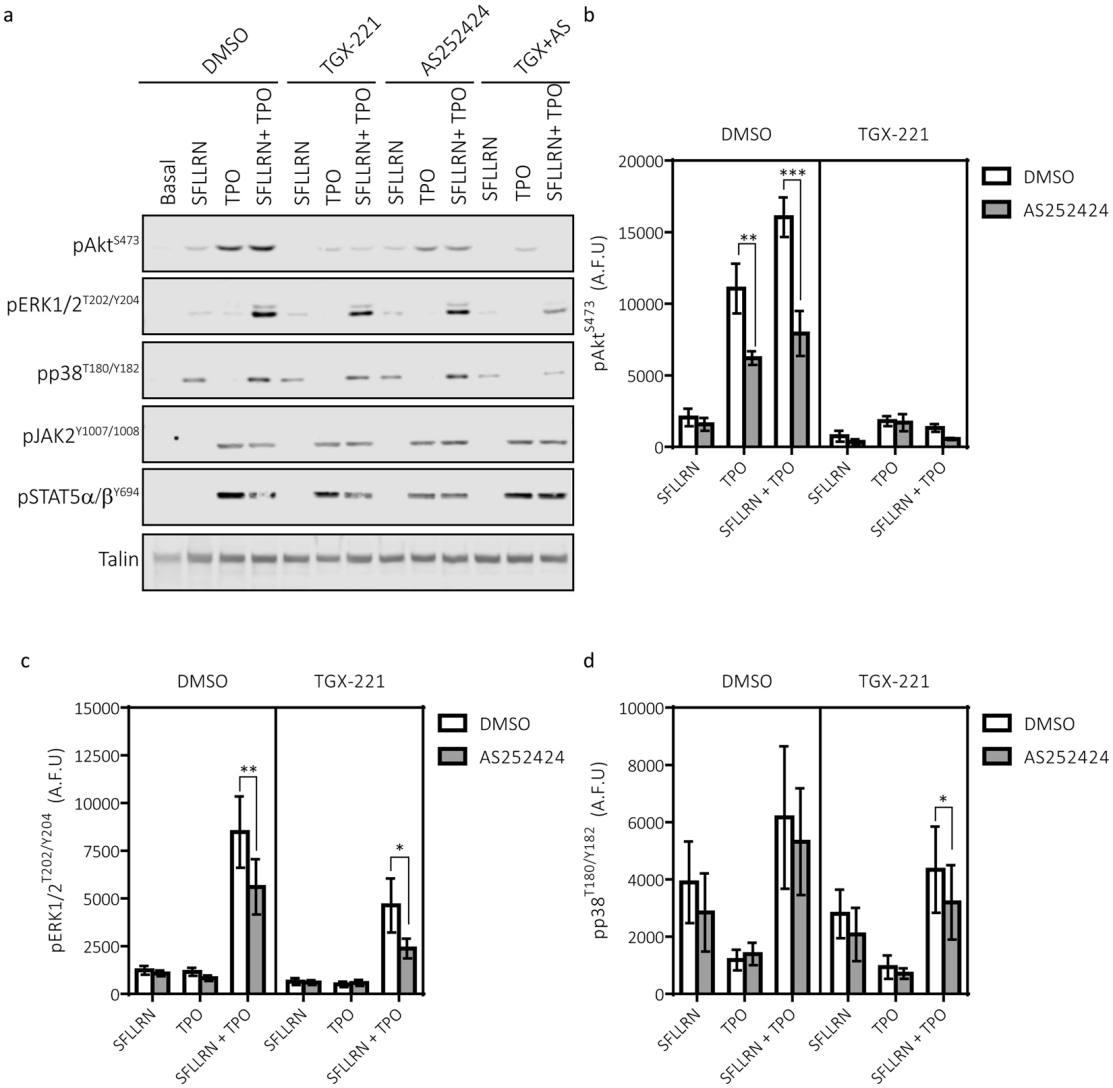
Role for p110γ in regulating the effect of TPO on platelets signalling. Washed human platelets were pre-incubated with TPO (100 ng/ml) for 5 min before stimulation with SFLLRN (1 μM, 5 min) in the absence or presence of the p110β inhibitor TGX-221 (200 nM, 15 min), p110γ inhibitor AS252424 (2 μM, 15 min) or both inhibitors. Platelets were lysed in 4x NuPAGE sample buffer containing 0.5M DTT and protein phosphorylation analysed by electrophoresis/immunoblotting. Samples were run on multiple gels for probing with multiple antibodies. For some experiments, gels and membranes were cut into two parts to be probed separately with different primary antibodies (e.g. pJAK2^Y1007/1008^ and pAkt^S473^, pSTAT5α/β^Y694^ and pERK^T202/Y204^). LI-COR® Image Studio (LI-COR®, Cambridge, UK) was used to create final images and to quantify bands as outlined in the Material and Methods. Full images and molecular weights from these experiments are presented in Supplementary Fig. S7. All samples in the panel figure shown are derived from the same donor/experiment. (**a**) Representative blot demonstrating that inhibition of either p110β and/or p110γ alters TPO induced signalling events. (**b**) AS252424 supressed TPO and SFLLRN + TPO induced phosphorylation of Akt^S473^ (n = 4 ± s.e.m). (**c**) AS252424 supressed the synergistic enhancement of ERK1/2^T202/Y204^ phosphorylation evoked by TPO. Furthermore, the suppression of ERK1/2^T202/Y204^ phosphorylation induced by pre-treating platelets with a combination of TGX-221 and AS252424 was greater than pre-treatment with TGX-221 alone (n = 4 ± s.e.m). (**d**) AS252424 alone did not alter phosphorylation of p38^T180/Y182^, however in combination with TGX-221 suppression of p38 phosphorylation induced by SFLLRN + TPO was observed (n = 5 ± s.e.m). Data are expressed as arbitrary fluorescence units (A.F.U, LI-COR® Image Studio). 2-way ANOVAs were performed with Bonferroni’s multiple comparisons test: (*Variable 1 = Stimulation, Variable 2 = Inhibitor). Significance was determined when p < 0.05, *p < 0.05, **p < 0.01, ***p < 0.001.

It was noted that pharmacological inhibition (Fig. 4a,c, Supplementary Fig. S1) or deletion of p110β (Supplementary Fig. S1) did not abolish the synergistic effect of TPO on ERK1/2 phosphorylation. Therefore, we speculated that an additional PI3K isoform may be involved in regulating this effect. Indeed, we found that pre-treatment of platelets with the p110γ inhibitor AS252424 resulted in a reduction of the synergistic effect of TPO on ERK1/2 phosphorylation (Fig. 5a,c). Furthermore, the combination of TGX-221 and AS252424 reduced ERK1/2 phosphorylation further than pre-treatment with TGX-221 alone (Fig. 5a,c), leaving little residual ERK1/2 phosphorylation. The latter may be independent of PI3K as pre-treatment of platelets with the PI3K inhibitor wortmannin reduced ERK1/2 phosphorylation to similar levels (Supplementary Fig. S3). These results demonstrate that both p110β and p110γ contribute to the synergistic effect of TPO on PAR-mediated ERK1/2 phosphorylation.

### Synergistic activation of ERK1/2 results in enhanced TxA_2_ generation

**Figure 6:**
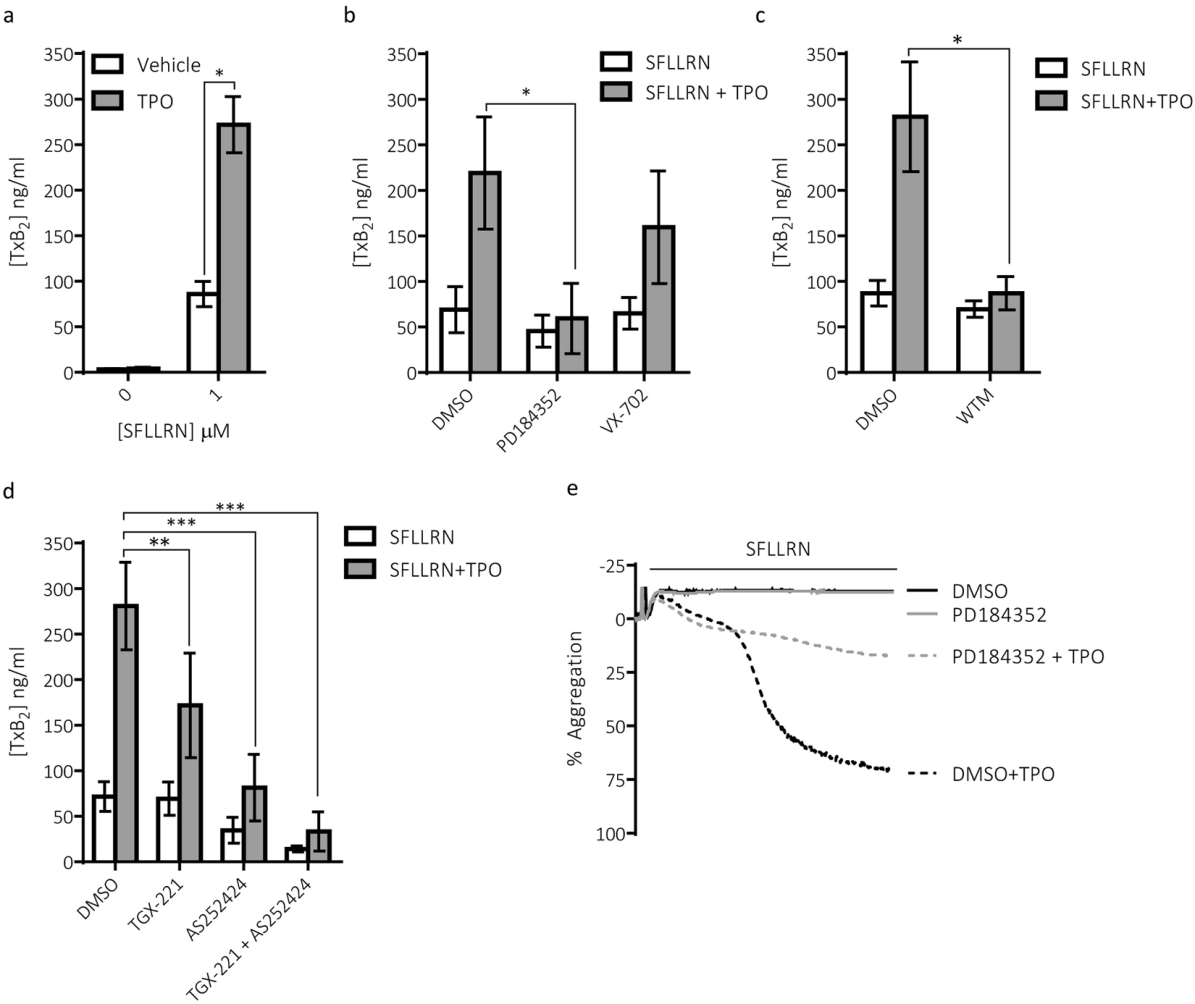
Role for ERK1/2 and PI3K in regulating TPO-induced increases in TxA_2_ generation. Human platelets (no indomethacin) were pre-incubated with TPO (100 ng/ml) for 5 min before stimulation with SFLLRN (1 μM, 5 min) in the absence or presence of the either the p38 inhibitor VX-702 (300 nM, 15 min), the MEK inhibitor PD184352 (300 nM, 15 min), the PI3K inhibitor wortmannin (100 nM, 15 min), the p110β inhibitor TGX-221 (200 nM, 15 min), the p110γ inhibitor AS242525 (2 μM, 15 min) or a combination of TGX-221 and AS242525. Supernatants were collected and TxB_2_ concentrations assessed using a colorimetric ELISA kit. (**a**) TPO synergistically enhanced PAR-mediated TxB_2_ production (n = 5 ± s.e.m). 2-way ANOVAs were performed with Bonferroni’s multiple comparisons test: (*Variable 1 = SFLLRN, Variable 2 = TPO). (**b**) This enhancement was supressed by the MEK inhibitor PD184352 but not the p38 inhibitor VX-702 (n = 3 ± s.e.m). 2-way ANOVAs were performed with Bonferroni’s multiple comparisons test: (*Variable 1 = Stimulation, Variable 2 = Inhibitor). (**c**) Furthermore, the enhancement in TxB_2_ production was dependent on PI3K as the pan-PI3K inhibitor wortmannin ablated the synergistic increase in TxB_2_ production evoked by SFLLRN and TPO (n = 7 ± s.e.m). 2-way ANOVAs were performed with Bonferroni’s multiple comparisons test: (*Variable 1 = Stimulation, Variable 2 = Inhibitor). (**d**) Both the p110β and p110γ PI3K isoforms were observed to contribute to TPO-mediated enhancements in TxB_2_ production. Both TGX-221 and AS252424 reduced TxB_2_ production and pre-treatment of platelets with both inhibitors resulted in further suppression of TxB_2_ production (n = 3 ± s.e.m). 2-way ANOVAs were performed with Bonferroni’s multiple comparisons test: (*Variable 1 = Stimulation, Variable 2 = Inhibitor). (**e**) Representative aggregation traces demonstrate that PD184352 can inhibit the secondary but not primary wave of aggregation induced by SFLLRN + TPO. Aggregation traces are representative of three independent experiments. Significance was determined when p < 0.05, *p < 0.05, **p < 0.01, ***p < 0.001.

ERK1/2 has previously been implicated in regulating the ability of TPO to prime platelet function via a TxA_2_-dependent pathway^3,31^. We therefore sought to confirm whether TPO can enhance TxA_2_ generation and if this enhancement is blocked under conditions where ERK1/2 phosphorylation is also attenuated. We confirmed that despite the inability of TPO to stimulate TxA_2_ generation by itself, it enhanced PAR-mediated TxA_2_ generation (Fig. 6a). Furthermore, this enhancement in TxA_2_ generation was blocked in the presence of the MEK inhibitor PD184352 but not the p38 inhibitor VX-702 (Fig. 6b). Under the conditions used, these inhibitors selectively blocked phosphorylation of ERK1/2 and the p38 substrates MAPKAPK2 and HSP27, respectively (Supplementary Fig. S4). We also demonstrate the vital role for PI3K in eliciting the ability of TPO to enhance TxA_2_ generation as pre-treatment of platelets with wortmannin ablated the priming effect of TPO (Fig. 6c). Moreover, pre-treatment with either the p110β inhibitor; TGX-221 or the p110γ inhibitor; AS252424 resulted in substantial reductions in SFLLRN + TPO-mediated TxA_2_ generation, with combined treatment largely blocking the effect (Fig. 6d). Interestingly, the p110γ inhibitor had a greater inhibitory effect than the p110β inhibitor, indicating that the p110γ isoform plays a more dominant role than the p110β isoform in regulating TxA_2_ generation. These results demonstrate an important role for p110β and p110γ in the synergistic effect of TPO on PAR-1 mediated ERK1/2 phosphorylation and thromboxane synthesis.

### ERK1/2 activation is important for the priming effect of TPO under conditions dependent on TxA_2_ generation

Our platelet functional studies were performed under conditions where TxA_2_ generation is inhibited (indomethacin-treated); demonstrating that TPO can enhance platelet function independently on TxA_2_ generation. However, previous studies have demonstrated that TPO can also enhance secondary aggregation and this is blocked by acetylsalicylic acid/aspirin^3^. We therefore hypothesised that in the absence of indomethacin, TPO-mediated enhancement of secondary platelet aggregation would be inhibited by PD184352. Indeed, when using sub-threshold concentrations of SFLLRN, TPO mediated two distinct waves of aggregation with the secondary but not primary wave being blocked by PD184352 (Fig. 6e).

## Discussion

**Figure 7:**
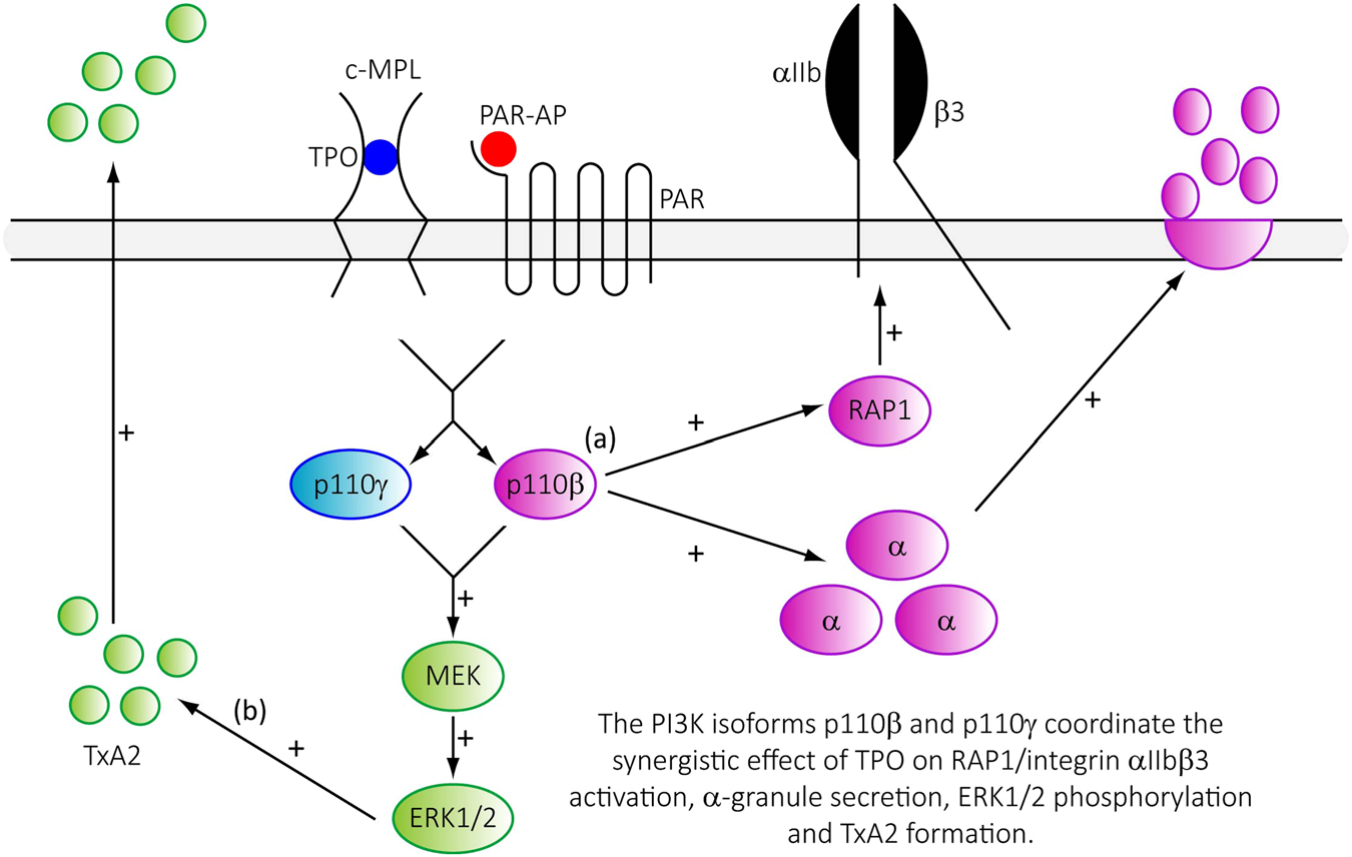
Schematic summarising how PI3K p110β and p110γ regulate TPO-mediated amplification of platelet function. Stimulation of platelets with the c-MPL agonist TPO enhances (+) PAR-mediated integrin α_IIb_β_3_ activation and α-granule secretion. This enhancement occurs with concurrent increases in the GTP-loading of the small GTPase RAP1, phosphorylation of ERK1/2 and subsequent TxA_2_ formation. These processes are regulated by the activation of the PI3K isoforms p110β and p110γ. With our data suggesting that (**a**) PI3K p110β is the dominant isoform in regulating integrin α_IIb_β_3_ activation and α-granule secretion whereas (**b**) PI3K p110γ is the dominant isoform in regulating the generation of TxA_2_.

This is the first study to demonstrate critical roles for the PI3K isoforms p110β and p110γ in supporting platelet priming by TPO. Using both pharmacological tools and genetic models we have found that p110β is the predominant PI3K isoform involved in mediating the priming effect of TPO on PAR-mediated integrin α_IIb_β_3_ activation and α-granule secretion, and the concurrent synergistic activation of RAP1 (Fig. 7). Furthermore, both p110β and p110γ play an essential role in the synergistic enhancement of PAR-mediated ERK1/2 phosphorylation and TxA2 synthesis (Fig. 7).

This and previous studies have demonstrated that TPO alone is unable to induce platelet functional responses, however when used in combination with other platelet agonists it can enhance aggregation, integrin α_IIb_β_3_ activation, granule secretion, TxA_2_ production and thrombus formation^32^. This is found to coincide with increases in the activation of pathways associated with both PI3K and MAPK signalling. In platelets binding of TPO to its receptor; c-MPL will elicit the activation of JAK2 ultimately leading to recruitment and tyrosine phosphorylation of SH2 domain containing STATs (signal transducer and activator of transcription) e.g. STAT3 and STAT5α/β^33,34^. Another event that occurs is the tyrosine phosphorylation of the p85 regulatory subunit of PI3K and its association with Gab1. This phosphorylation of p85 relieves its inhibitory activity on the p110 catalytic subunits resulting in an increase in PI3K activity and the phosphorylation of downstream effectors such as Akt. It is this increase in PI3K activity that is a key driver in enhancing PAR-mediated integrin activation and α-granule by TPO, with a likely role for the small GTPase RAP1 in this process. More specifically, based on our findings with both pharmacological inhibitors and genetic models, it is an increase in the activity of the p110β isoform of PI3K that mediates this TPO effect. In keeping with this observation, the phosphorylation of the PI3K effector Akt is considerably reduced by either treating platelets with TGX-221 or by knocking out p110β. However, residual phosphorylation when stimulating with TPO alone or TPO and a PAR agonist does remain. This contrasts with our findings with the pan-PI3K inhibitor wortmannin, indicating that TPO signals through multiple PI3K isoforms. Our findings suggest that c-MPL activation signals to a second PI3K isoform; p110γ. The evidence for this is that i) the p110γ inhibitor reduces TPO-mediated Akt phosphorylation and ii) the combination of p110β and p110γ inhibition ablates TPO-mediated Akt phosphorylation. Furthermore, deletion of the p110α isoform and p110δ inhibitors did not alter TPO-mediated phosphorylation of Akt (data not shown). The finding that TPO can signal and promote functional responses via p110γ is intriguing and unexpected. Activation of this PI3K isoform is typically thought to occur downstream of GPCRs, via the Gβγ subunits, with general opinion suggesting that p110γ is essential for GPCR but not tyrosine-kinase associated receptor, mediated activation of PI3K^35,36^. However, our data clearly shows that TPO, which signals through the cytokine receptor c-MPL, can enhance ERK phosphorylation and TxA_2_ synthesis through PI3K p110γ. Interestingly, there have been other reports of p110γ playing an important functional role downstream of other tyrosine-kinase associated receptors. A study by Alcázar *et al*.^37^, demonstrated that p110γ was activated by T cell receptor (TCR) ligation and that deletion of p110γ resulted in impairments in TCR signalling and T cell activation. Furthermore, Schmid *et al*.^36^, demonstrated a role for p110γ in regulating tumour inflammation initiated by a range of ligands coupling to receptor tyrosine kinases; VEGF-A, toll like/interleukin-1 receptors; IL-1β and cytokine receptors; IL-6.

In addition to PI3K, TPO binding to c-MPL stimulates the activation of at least two MAPK pathways; ERK1/2 and p38. This is mediated by the recruitment and tyrosine phosphorylation of Grb2, SHC, and SOS, and GTP loading of the small GTPase RAS. In haematopoietic stem cells and megakaryocytes this results in promotion of proliferation and differentiation^38–40^. However, in platelets TPO binding c-MPL doesn’t result in the phosphorylation of either ERK1/2 or p38^41–43^. This is despite platelets expressing the requisite upstream signalling molecules and TPO being able to stimulate the activation of RAS^42^. One explanation for this is that in platelets, RAS activation is not sufficient for ERK activation, as activation of PKC is required for the activation of ERK at the level of/upstream of MEK^42^. However, despite the inability of TPO to induce phosphorylation/activation of MAPKs, it can subtly enhance phosphorylation of p38 and induce a striking highly synergistic enhancement in ERK1/2 phosphorylation. With the ability of TPO to induce these enhancements being not only dependent on the PI3K isoform p110β but also p110γ.

In human platelets, the MAPK family members have been implicated in regulating the production of TxA_2_; a prothrombotic agent which activates and amplifies platelet responses through the thromboxane receptors. TxA_2_ is generated by thromboxane synthase from prostaglandin H2 (PGH_2_) which is itself generated from arachidonic acid by the aspirin target; cyclooxygenase. Previous work attributes the MAPK family members in being able to regulate TxA_2_ production through their ability to phosphorylate and therefore increase the catalytic activity of cytosolic phospholipase A2 (cPLA_2_). This enzyme in turn will hydrolyse various phospholipids, into arachidonic acid. Our and previous findings clearly demonstrate that TPO can markedly enhance TxA_2_ and, in agreement with van Willigen *et al*.,^31^ we believe this is mediated by the enhanced activation of ERK1/2. This is based on our finding that the MEK1/2 inhibitor PD184352, which blocks ERK1/2 phosphorylation/activation but not p38 activity, ablates the synergistic component of TxA_2_ generation induced by TPO and SFLLRN. Furthermore, the p38 inhibitor; VX-702, which specifically blocked the phosphorylation of known p38 substrates, but not the phosphorylation of ERK1/2 or p38 itself, did not ablate the synergistic generation of TxA_2_.

In support of our findings regarding ERK1/2 phosphorylation, TxA_2_ generation induced by the combination of SFLLRN and TPO was drastically reduced by pan-PI3K inhibition. This reduction was an ablation of the synergistic component of TxA_2_ generation, as SFLLRN and TPO-mediated TxA_2_ generation in the presence of wortmannin was almost identical to TxA_2_ generation mediated by SFLLRN alone. In a further mirroring of our results on ERK1/2 phosphorylation, both the p110β and p110γ inhibitors could reduce the synergistic component and a further reduction was observed with combined inhibition. These data highlight and strengthen the idea that TPO through the PI3K isoforms; p110β and p110γ can synergistically increase SFLLRN-mediated activation of ERK1/2 which in turn leads to an enhancement in TxA_2_ production. Furthermore, this enhancement in TxA_2_ can contribute to a pro-aggregatory response under conditions where cyclooxygenase is not directly inhibited.

We therefore conclude that TPO through the PI3K isoforms p110β and p110γ can enhance platelet activation through TxA_2_ independent and dependent pathways (Fig. 7). Therefore, under conditions where TPO levels or c-MPL signalling are pathophysiologically elevated, the pro-aggregatory response may be dampened by traditional anti-platelet therapies or ablated by PI3K inhibition.

## Materials and Methods

### Materials

Protease activated receptor 1 (PAR-1)-activating peptide (SFLLRN-NH_2_) was from Bachem (Bubendorf, Switzerland). Protease activated receptor 4 (PAR-4)-activating peptide (AYPGKF-NH2) was from PeptideSynthetics (Peptide Protein Research Ltd, Hampshire, UK). Recombinant human TPO and wortmannin were from Bio-Techne (Abingdon, UK). Recombinant murine thrombopoietin (TPO) was from PeproTech (London, UK). TGX-221, AS-252424, PD184352, IC-87114, PIK-75 and VX-702 were from Selleckchem (Stratech Scientific Ltd, Ely, UK). Mouse anti-human PAC-1 conjugated to FITC and mouse anti-human CD62P (AK-4) conjugated to PE were from BD Biosciences (Oxford, UK). Rat anti-mouse JON/A conjugated to PE and rat anti-mouse CD62P (Wug.E9) conjugated to FITC were from emfret ANALYTICS (Eibelstadt, DE). Phospho-ERK1/2^T202/Y204^ (#9101), phospho-Akt^S473^ (#4060), phospho-JAK2^Y1007/1008^ (#3771), phospho-STAT5α/β^Y694^ (#4322), phospho-p38^T180/Y182^ (#4511), phospho-MAPKAPK2^T334^ (#3007), phospho-HSP27^S78^(#2405), ERK1/2 (#9102), Akt (#2920), and p110β (#3011) antibodies were from Cell Signaling Technology (New England Biolabs, Hitchin, UK). Talin (#SC7534), GAPDH (#SC25778) and RAP1 (#SC65) antibodies were from Santa Cruz Biotechnology (Insight Scientific, Middlesex, UK). Odyssey blocking buffer (TBS) was from LI-COR Biosciences (Cambridge, UK). Secondary antibodies were from Jackson ImmunoResearch (Stratech Scientific Ltd, Ely, UK). TxB_2_ ELISA kit was from Enzo Life Sciences (Exeter, UK). All other materials were from Sigma Aldrich (Poole, UK).

### Human studies

Venous blood was drawn into a syringe containing 4% trisodium citrate (1:9, v/v). Blood was obtained with approval from the local Research Ethics Committee of the University of Bristol from healthy drug-free volunteers, who gave full informed consent in accordance with the Declaration of Helsinki.

### Mouse Models

All animal studies were approved by the local research ethics committee at the University of Bristol, UK and mice were bred and maintained for this purpose under the UK Home Office licence PPL30/3445. Mice (8–24 weeks old) were sacrificed by rising CO_2_ inhalation, in accordance with Schedule 1 of the Animals (Scientific Procedures) Act (1986), and blood was drawn by cardiac puncture into a syringe containing 4% trisodium citrate (1:9, v/v). For transgenic experiments appropriate wild-type (WT) controls were used (strain, age and sex matched). p110α flox/flox:Pf4-Cre mice (C57BL/6) and p110β mice (mixed C57BL/6J x 129 Sv) have been previously described^21,44^.

### Platelet Isolation

Washed human and mouse platelets were prepared as previously described^21,45^. Platelets were resuspended in HEPES–Tyrode’s buffer (pH 7.2; 145 mM NaCl, 5 mM KCl, 0.5 mM Na_2_HPO_4_, 1 mM MgSO_4_, 10 mM HEPES), supplemented with 0.1% glucose (w/v), 10 μm indomethacin and 0.02 U mL^−1^ apyrase.

### Platelet Function Assays

Activation of integrin α_IIb_β_3_ and α-granule secretion were assessed by flow cytometry as previously described using a BD Accuri^TM^ C6 plus (BD Biosciences, Oxford, UK)^46^. For human platelets a FITC-conjugated antibody directed against the high affinity form of integrin αIIbβ3 (PAC-1) and PE-conjugated antibody for the α-granule marker CD62P (AK-4) were used. For mouse platelets a PE-conjugated antibody directed against the high affinity form of integrin α_IIb_β_3_ (JON/A) and a FITC-conjugated antibody for the α-granule marker CD62P (Wug.E9) were used. For platelet aggregation, platelets (2 × 10^8^/mL in the absence of indomethacin) were pre-incubated with inhibitors/TPO as indicated before agonist-stimulated aggregation was monitored using a CHRONO-LOG® Model 700 aggregometer at 37 °C, with continuous stirring at 1000 rpm. Data were recorded using Aggrolink Version 8 software (CHRONO-LOG®).

### Protein Extraction and Immunoblotting

Washed platelets (4 × 10^8^/mL) were stimulated as indicated and lysed directly in 4X NuPAGE sample buffer containing 0.5 M dithiothreitol (DTT). Proteins were resolved by electrophoresis as previously described^45^ and transferred onto polyvinylidene fluoride (PVDF) membranes. For some experiments, gels and membranes were cut into two parts to be probed separately with different primary antibodies^47^. Proteins were visualized by near-infrared detection using a LI-COR® Odyssey imaging system (providing a wide linear dynamic range) unless indicated otherwise. LI-COR® Image Studio (LI-COR®, Cambridge, UK) was used to create final images, the software analysis options only affect how raw data pixels are mapped to the screen and does not alter the experimental data. LI-COR® Image Studio (LI-COR®, Cambridge, UK) was used to quantify bands. Bands were defined using the rectangle shape tool to obtain fluorescence values and median local background (intensity of pixels in a border around the shape) was automatically subtracted.

### RAP1 Activation Assay

Was performed using the Ral guanine nucleotide dissociation stimulator RAP-binding domain protein (GST-RALGDS-RBD) as previously described^13^.

### TxA_2_ Generation

TxA_2_ generation was assessed by measuring TxB_2_ in platelet supernatants using a commercially available colorimetric ELISA kit (Enzo Life Sciences, Exeter, UK) as previously described^48^. Briefly, platelets (4 × 10^8^/ml in the absence of indomethacin) were stimulated as indicated before addition of 5 mM ethylenediaminetetraacetic acid and 200 μM indomethacin. Platelets were pelleted (4 min, 12,000 × g) and supernatant collected for analysis.

### Data Analysis

Data were analysed and fitted using GraphPad Prism 7 software. All data are presented as the mean ± s.e.m of at least three independent observations. Concentration-response curves were fitted with a four-parameter logistic equation.

$$Y={\rm{Bottom}}+({\rm{Top}}-{\rm{Bottom}})/(1+{10}^{((\mathrm{Log}EC50-X)\ast {\rm{HillSlope}})}).$$

Concentration-response curves from different donors were pooled after normalising them to the maximal response (Top) obtained in the vehicle-control samples. Data presented with statistical analysis were tested as indicated in the figure legends (GraphPad Prism 7).

## Supplementary information

Supplementary Figures

## Data Availability

The datasets generated and analysed during the current study are available on reasonable request.
