# Supplementary material for: Critical roles for the phosphatidylinositide 3-kinase isoforms p110β and p110γ in thrombopoietin-mediated priming of platelet function
Source: Sci Rep. 2019 Feb 6;9:1468. doi: 10.1038/s41598-018-37012-9 (PMC6365529; doi:10.1038/s41598-018-37012-9)
Supplement: Supplementary file 1 — Supplementary Figures [file 41598_2018_37012_MOESM1_ESM.pdf]

Critical roles for the phosphatidylinositide 3-kinase isoforms p110β and p110γ in thrombopoietin-mediated priming of platelet function

Samantha F. Moore *, Nina R. Smith *, Thomas A. Blair *, †, Tom N. Durrant *, Ingeborg Hers * ‡.

* School of Physiology, Pharmacology and Neuroscience, Biomedical Sciences Building, University of Bristol, BS8 1TD, UK, †present address: Harvard Medical School and Boston Children's Hospital, Division of Hematology/Oncology, Boston, Massachusetts, United States.

‡Address correspondence to: Dr Ingeborg Hers, School of Physiology, Pharmacology and Neuroscience, Biomedical Sciences Building, University of Bristol, Bristol, BS8 1TD, United Kingdom. Tel: 0044 117 331 2191, Fax: 0044 117 331 2288, E-mail: i.hers@bristol.ac.uk.

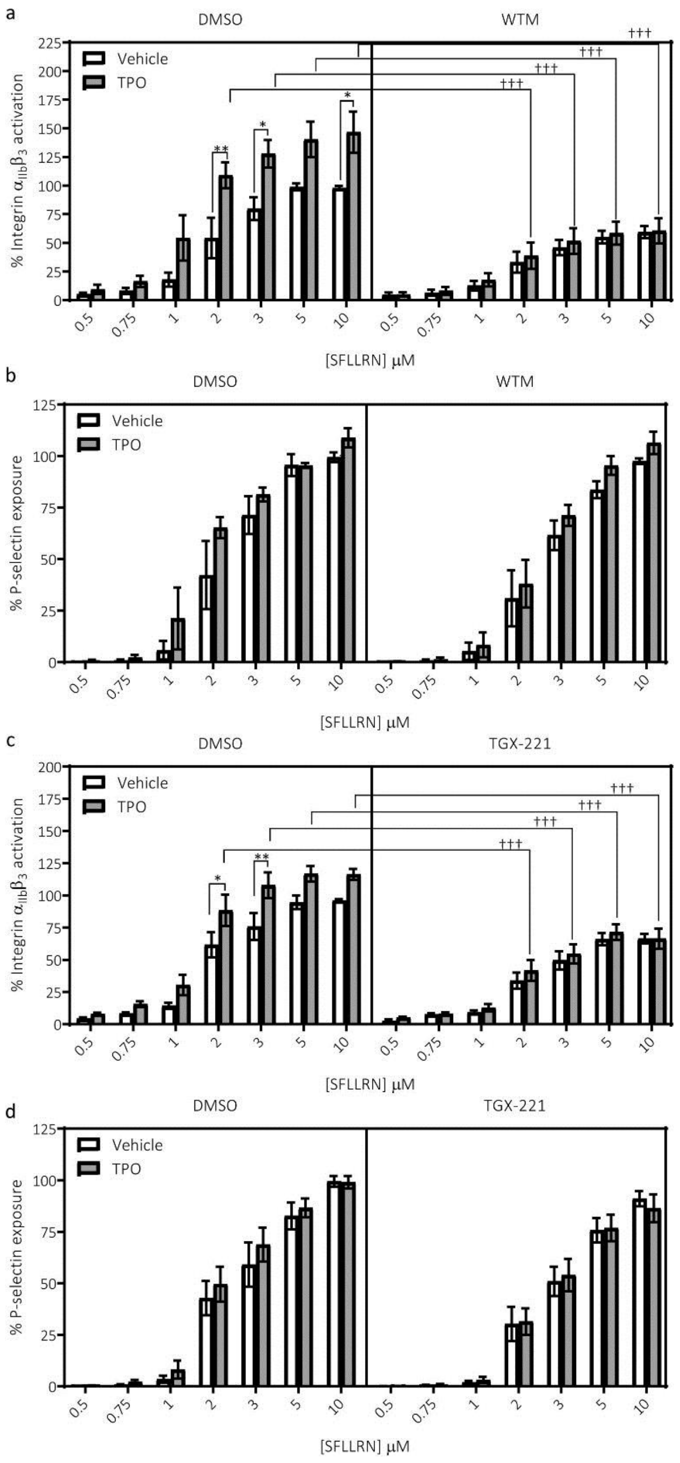

Supplementary Figure S1: Histograms of “Figure 1 Enhancement of platelet function by TPO is supressed by the pan-PI3K inhibitor wortmannin and the p110β selective inhibitor TGX-221”. (a-b) Effect of TPO (100 ng/ml) on SFLLRN-mediated integrin $\alpha_{IIb}\beta_3$ activation and α-granule secretion in the presence of the pan-PI3K inhibitor wortmannin (WTM, 100 nM, 15 min). **(c-d)** Effect of TPO (100 ng/ml) on SFLLRN-mediated integrin $\alpha_{IIb}\beta_3$ activation and α-granule secretion in the presence of the PI3K p110β selective inhibitor TGX-221 (200 nM, 15 min). Statistical testing to determine differences between treatment groups at each concentration of SFLLRN was performed using 2-way ANOVAs with Bonferroni's multiple comparisons test: (*Variable 1 = SFLLRN concentration, Variable 2 = TPO or †Variable 1 = SFLLRN concentration, Variable 2 = Inhibitor). Significance was determined when $p<0.05$, *$p<0.05$, **$p<0.01$, ***$p<0.001$.

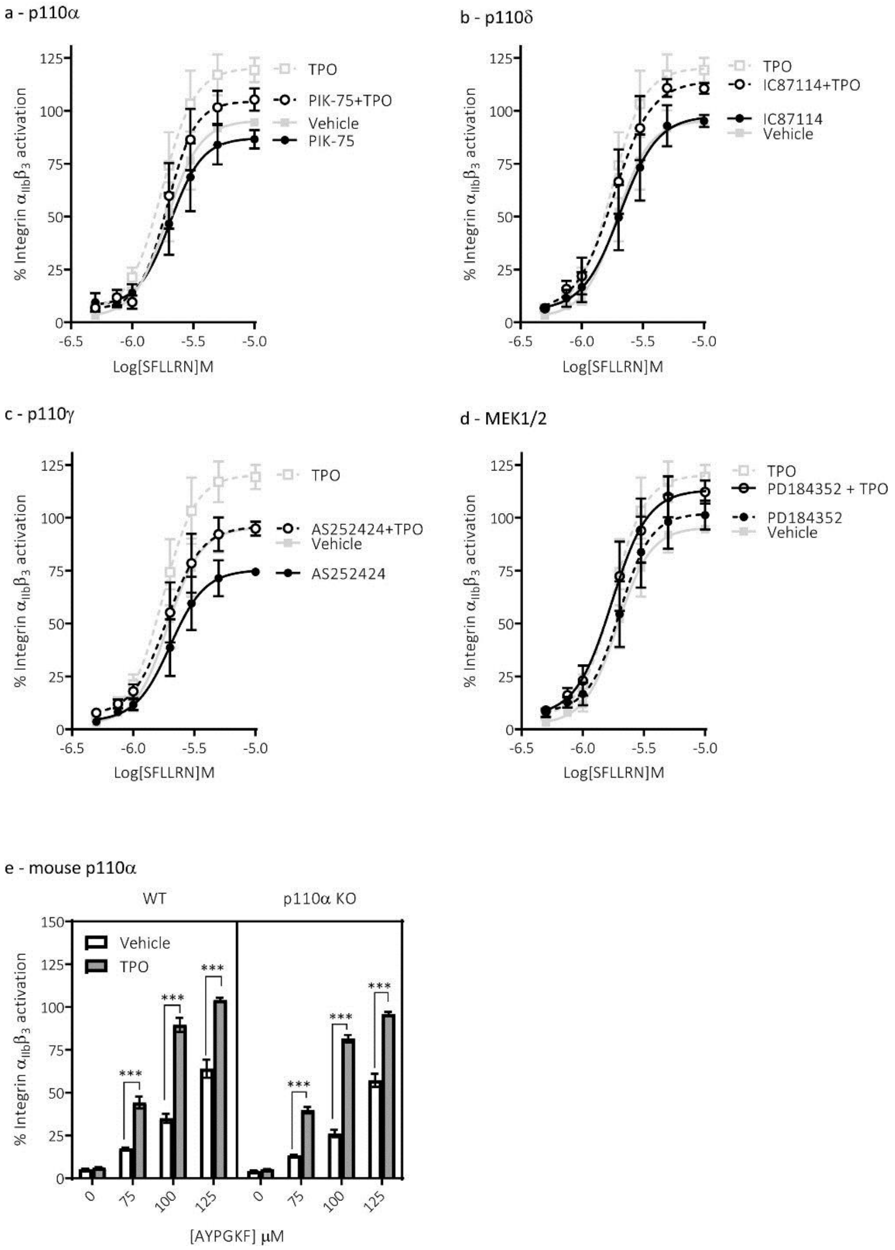

Supplementary Figure S2: Examining the role of the PI3K isoforms p110α, p110δ and p110γ in TPO-mediated enhancement in integrin $\alpha_{IIb}\beta_3$ activation. SFLLRN-mediated integrin $\alpha_{IIb}\beta_3$ activation was enhanced by TPO (100 ng/ml, 5 min), resulting in a left shift of the curve (pEC50 = 5.66 ± 0.09 to 5.71 ± 0.09, n = 5 ± s.e.m, p<0.01). TPO was able to enhance SFLLRN-mediated integrin $\alpha_{IIb}\beta_3$ activation in the presence of the **(a)** p110α selective inhibitor PIK-75 (50 nM, 15 min - pEC50 = 5.63 ± 0.09 to 5.69 ± 0.1, n = 5 ± s.e.m, p<0.001), **(b)** p110δ selective inhibitor IC87114 (1 μM, 15 min - pEC50 = 5.72 ± 0.08 to 5.80 ± 0.08, n = 4 ± s.e.m, p<0.001), **(c)** p110γ selective inhibitor AS252424 (2 μM, 15 min - pEC50 = 5.62 ± 0.1 to 5.67 ± 0.09, n = 5 ± s.e.m, p<0.01) or **(d)** MEK1/2 inhibitor PD184352 (300 nM, 15 min - pEC50 = 5.66 ± 0.1 to 5.71 ± 0.1, n = 5 ± s.e.m, p<0.01). Curves were fitted for each individual donor and curve parameters calculated using a four-parameter logistic equation (GraphPad Prism 7.0). For displayed graphs, concentration-response curves from different donors were pooled after normalising them to the maximal response (curve top) obtained in the vehicle-control samples. Statistical testing of pEC_{50} values was performed using 2-way ANOVAs with Bonferroni's multiple comparisons test: (Variable 1 = TPO, Variable 2 = Inhibitor). Significance was determined when p<0.05. **(e)** In support of the findings with PIK-75, deletion of p110α in mouse platelets did not supress the effect of TPO on AYPGKF-mediated integrin $\alpha_{IIb}\beta_3$ activation (n = 10 ± s.e.m). 2-way ANOVAs were performed with Bonferroni's multiple comparisons test: (*Variable 1 = concentration of AYPGKF, Variable 2 = TPO). Significance was determined when p<0.05, *p<0.05, **p<0.01, ***p<0.001.

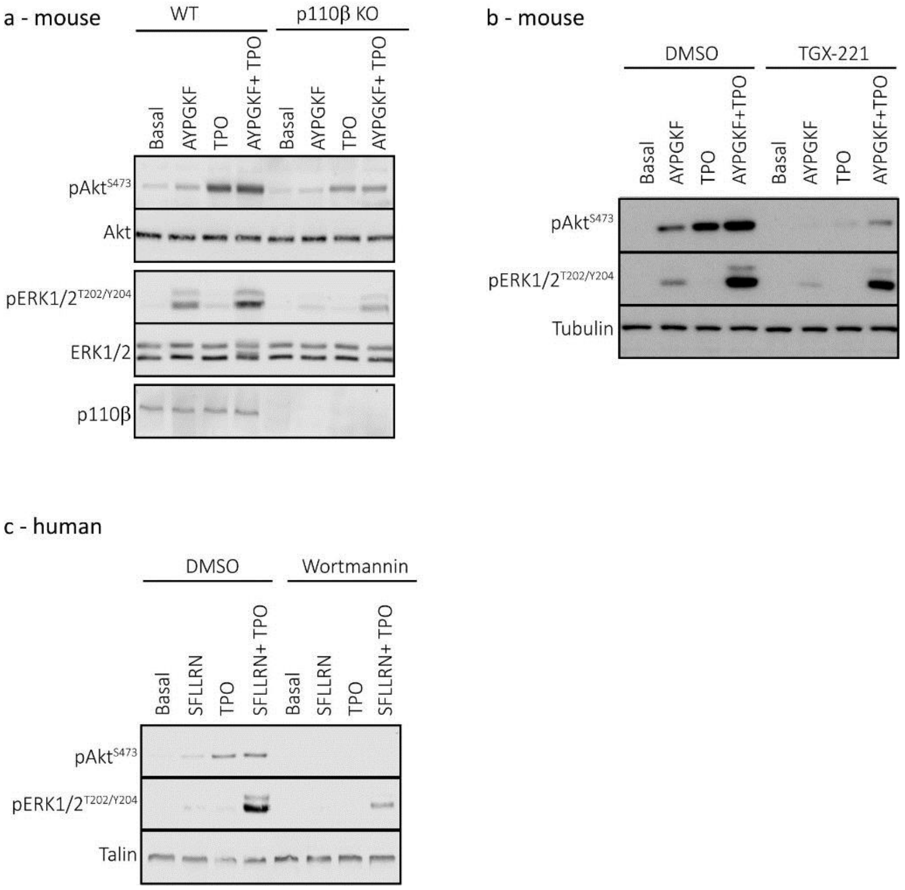

Supplementary Figure S3: Role of PI3K/p110β in TPO-mediated synergistic phosphorylation of pERK1/2$^{T202/Y204}$. Washed platelets stimulated as indicated were lysed in 4x NuPAGE sample buffer containing 0.5M DTT and protein phosphorylation analysed by electrophoresis/immunoblotting. Samples were run on multiple gels for probing with multiple antibodies. For some experiments, gels and membranes were cut into two parts to be probed separately with different primary antibodies (e.g. p110β/talin and pERK$^{T202/Y204}$). Phospho-blots were stripped for reprobing with total Akt and total ERK antibodies. LI-COR® Image Studio (LI-COR®, Cambridge, UK) was used to create final images and to quantify bands as outlined in the Material and Methods, except for S.Fig1b where HRP (horseradish peroxidase)/chemiluminescence was used. All samples in each panel figure shown are derived from the same mouse/donor/experiment. **(a)** Representative blot demonstrating that TPO can enhance phosphorylation of Akt^{S473} and ERK1/2$^{T202/Y204}$ in mouse platelets, but this enhancement is supressed in platelets lacking the p110β PI3K isoform. **(b)** Representative blot demonstrating that TPO can enhance phosphorylation of Akt and ERK1/2$^{T202/Y204}$ in mouse platelets, but this enhancement is supressed in platelets pre-treated with TGX-221. **(c)** Representative blot demonstrating that wortmannin supressed TPO's synergistic effect on the phosphorylation of ERK1/2$^{T202/Y204}$, leaving little residual phosphorylation. Blots are representative of three/four independent experiments.

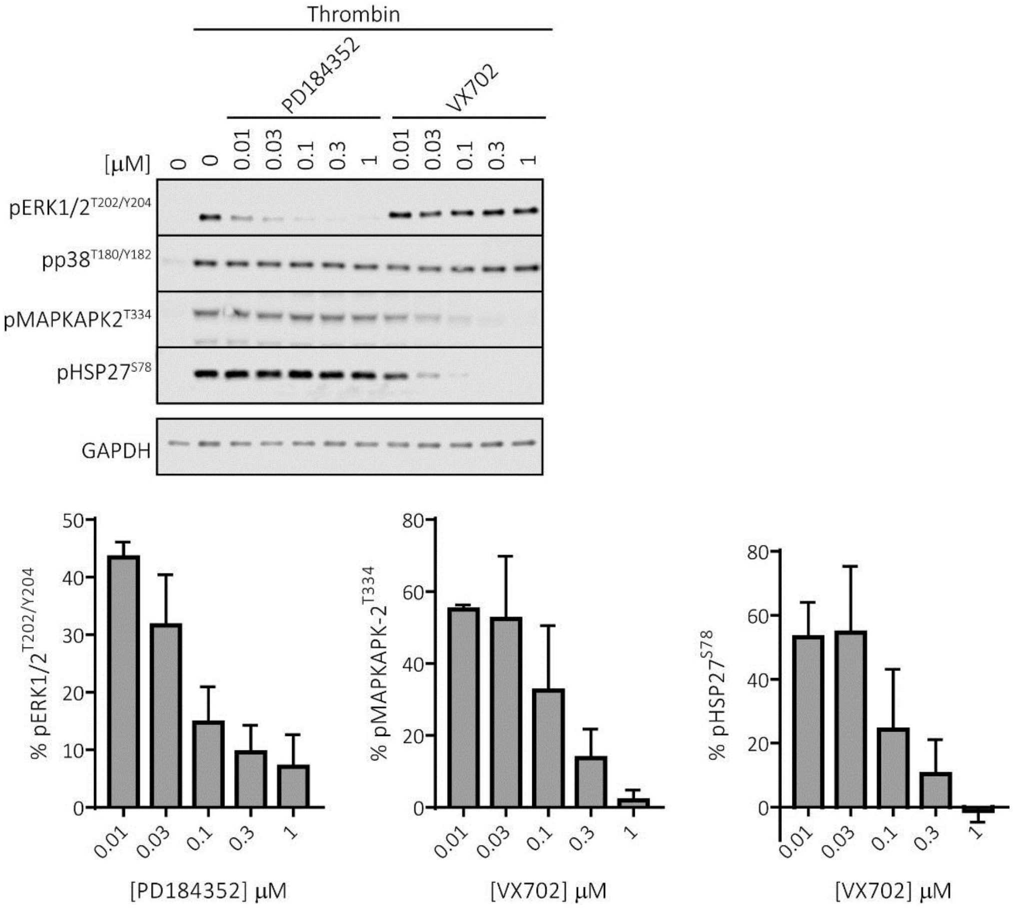

Supplementary Figure S4: **Effect of PD184352 and VX-702 on MAPK activation.** Washed human platelets were stimulated with thrombin (0.2 U/ml, 2 min) in the absence or presence of the MEK inhibitor PD184352 or p38 inhibitor VX-702 (15 min). Platelets were lysed in 4x NuPAGE sample buffer containing 0.5 M DTT and protein phosphorylation analysed by electrophoresis/immunoblotting. Samples were run on multiple gels for probing with multiple antibodies. LI-COR® Image Studio (LI-COR®, Cambridge, UK) was used to create final images and to quantify bands as outlined in the Material and Methods. All samples in the panel figure shown are derived from the same donor/experiment. Representative blot and histograms demonstrating that PD1843252 can concentration dependently inhibit the phosphorylation of ERK1/2$^{T202/Y204}$ but not MAPKAPK2^{T334} or HSP27^{S78} in contrast to VX-702. Blots are representative of three independent experiments. Histogram is n = 3 ± s.e.m.

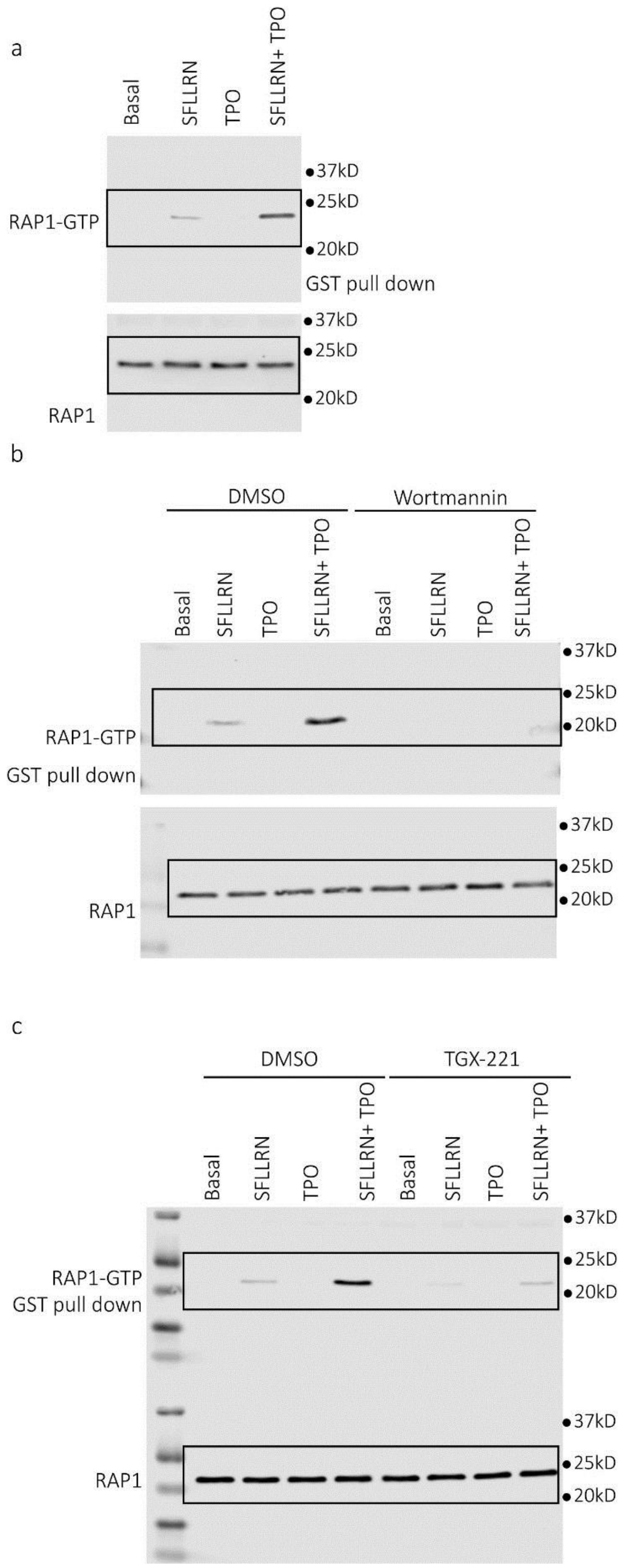

Supplementary Figure S5: **Full blots for "Figure 3: Critical role for PI3K p110β in the synergistic activation of the small GTPase RAP1"**

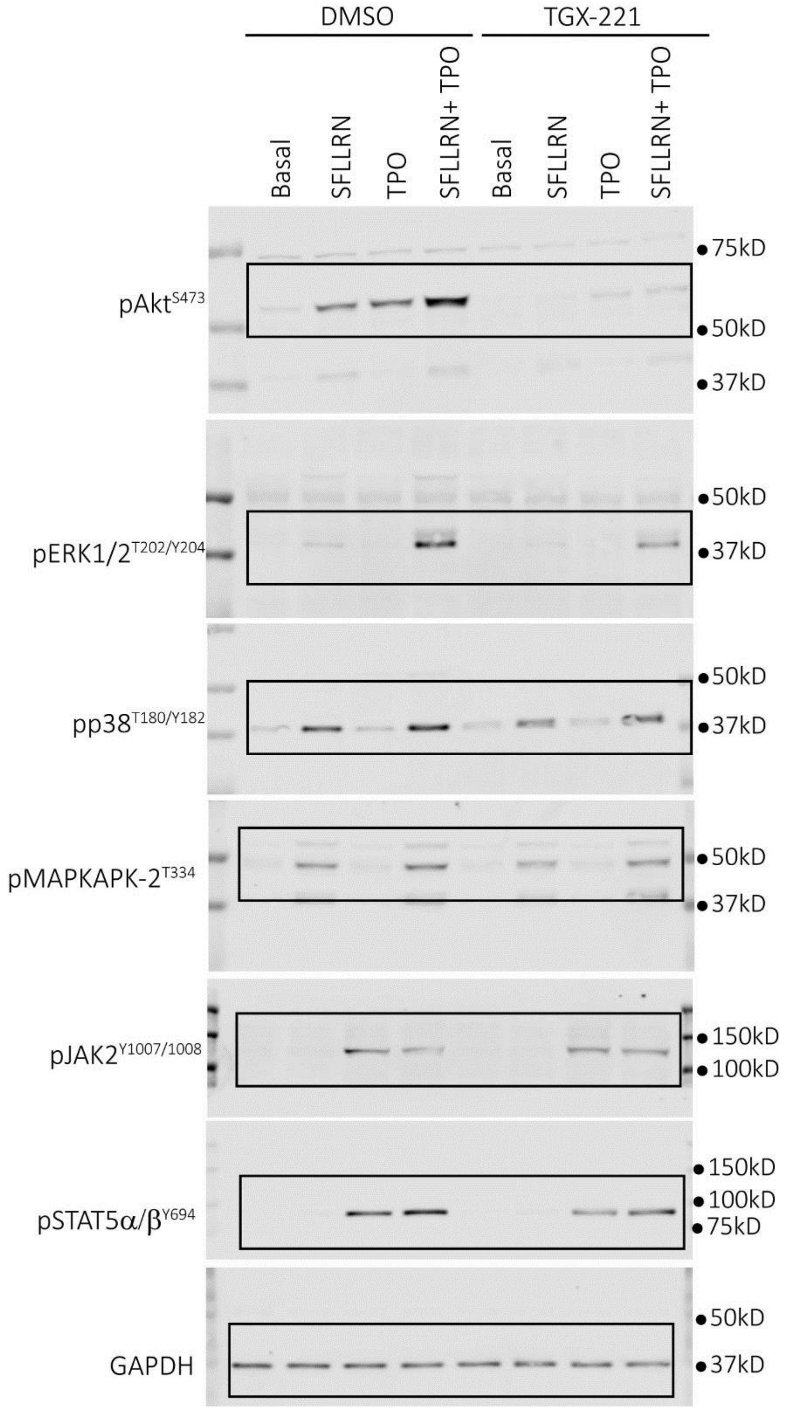

Supplementary Figure S6: **Full blots for "Figure 4: TPO enhances SFLLRN-mediated signalling events"**

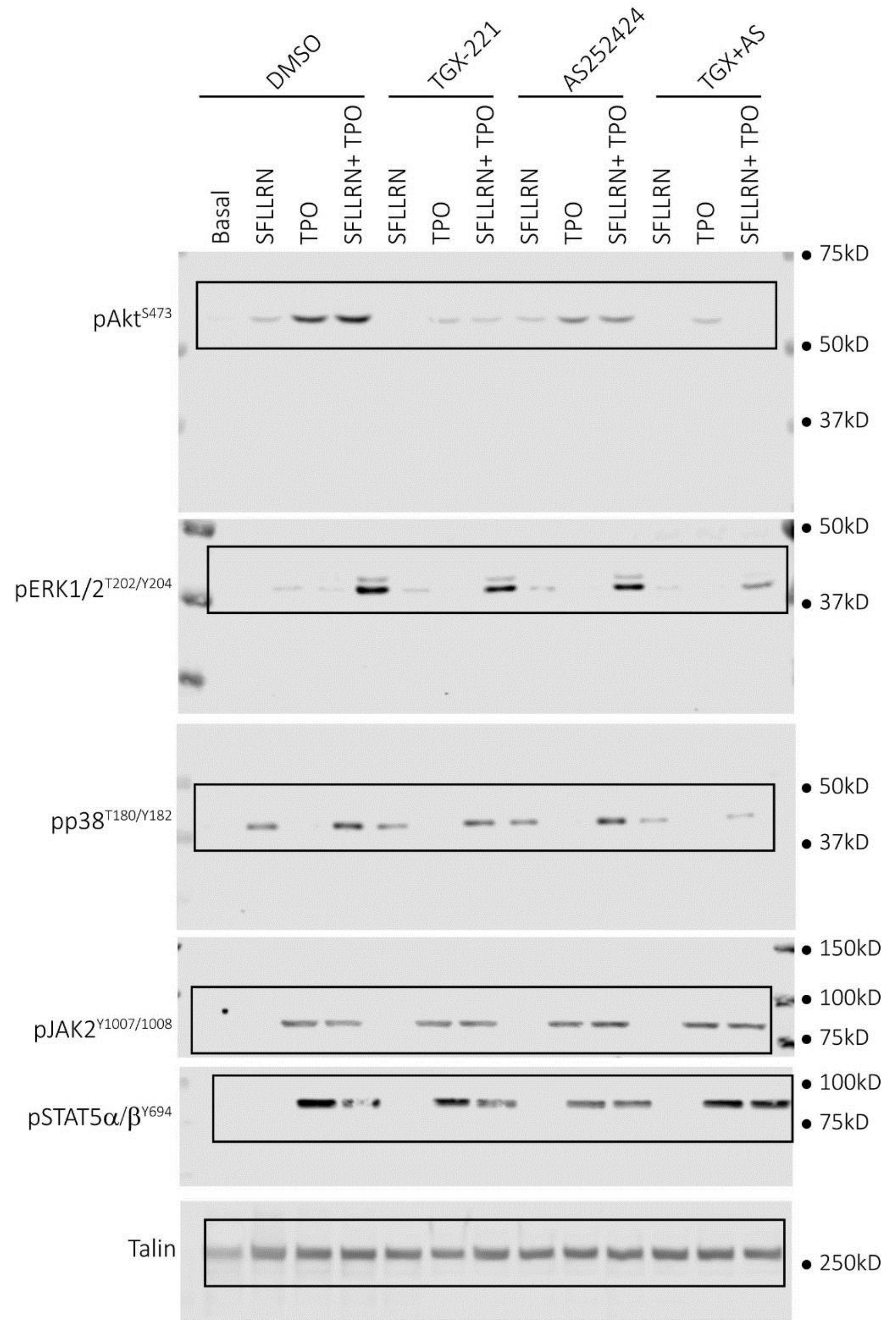

Supplementary Figure S7: **Full blots for "Figure 5: Role for p110γ in regulating the effect of TPO on platelets signalling"**
